# Single-Cell Microwell Platform Reveals Circulating Neural Cells as a Clinical Indicator for Patients with Blood-Brain Barrier Breakdown

**DOI:** 10.34133/2021/9873545

**Published:** 2021-07-08

**Authors:** Yu Zhang, Antony R. Warden, Khan Zara Ahmad, Yanlei Liu, Xijun He, Minqiao Zheng, Xinlong Huo, Xiao Zhi, Yuqing Ke, Hongxia Li, Sijia Yan, Wenqiong Su, Deng Cai, Xianting Ding

**Affiliations:** ^1^State Key Laboratory of Oncogenes and Related Genes, Institute for Personalized Medicine, School of Biomedical Engineering, Shanghai Jiao Tong University, 1954 Huashan Road, Shanghai 200030, China; ^2^Shanghai Engineering Research Centre for Intelligent Diagnosis and Treatment Instrument, Department of Instrument Science and Engineering, School of Electronic Information and Electrical Engineering, Shanghai Jiao Tong University, 800 Dongchuan Road, Shanghai 200240, China; ^3^Department of Neurosurgery, Wenling Hospital Affiliated to Wenzhou Medical University, Chuan'an Nan Road, Chengxi Subdistrict, Wenling, 317500 Zhejiang, China; ^4^Central Laboratory, Wenling Hospital Affiliated to Wenzhou Medical University, Chuan'an Nan Road, Chengxi Subdistrict, Wenling, 317500 Zhejiang, China; ^5^Department of Neurology, Wenling Hospital Affiliated to Wenzhou Medical University, Chuan'an Nan Road, Chengxi Subdistrict, Wenling, 317500 Zhejiang, China; ^6^Department of Thoracic Surgery, Shanghai Chest Hospital, Shanghai Jiao Tong University, 241 West Huaihai Road, Shanghai 200030, China

## Abstract

Central nervous system diseases commonly occur with the destruction of the blood-brain barrier. As a primary cause of morbidity and mortality, stroke remains unpredictable and lacks cellular biomarkers that accurately quantify its occurrence and development. Here, we identify NeuN^+^/CD45^−^/DAPI^+^ phenotype nonblood cells in the peripheral blood of mice subjected to middle cerebral artery occlusion (MCAO) and stroke patients. Since NeuN is a specific marker of neural cells, we term these newly identified cells as circulating neural cells (CNCs). We find that the enumeration of CNCs in the blood is significantly associated with the severity of brain damage in MCAO mice (*p* < 0.05). Meanwhile, the number of CNCs is significantly higher in stroke patients than in negative subjects (*p* < 0.0001). These findings suggest that the amount of CNCs in circulation may serve as a clinical indicator for the real-time prognosis and progression monitor of the occurrence and development of ischemic stroke and other nervous system disease.

## 1. Introduction

Central nervous system diseases, including brain injury, stroke [1, 2], brain tumors [3, 4], neurodegenerative diseases [5], and cognitive dysfunction associated with aging [6, 7], commonly occur via the destruction of the blood-brain barrier (BBB). In stroke, for example, BBB breakdown is observed in the venous microvessels and distal capillaries at 0.5-2 h within middle cerebral artery occlusion (MCAO) [8, 9]. Eighty-six percent of all cases of stroke are ischemic in nature [10]. The identification of ischemia, which is mainly diagnosed through clinical examination and neuroimaging, is heavily dependent on local availability [11, 12]. As a primary cause of morbidity and mortality, stroke lacks effective cellular biomarkers that accurately quantify its occurrence and development. Therefore, biomarkers that signal and qualify the risk of initial or recurrent acute stroke are important [13]. Circulating cells secreted by organs into the circulatory system act as molecular biomarkers (e.g., proteins, microRNAs, and cell-free nucleic acids) that provide critical information regarding health and illness [14].

Circulating cells are ideal biomarkers, which can provide noninvasive diagnoses, prognoses, and therapy guidance. New nonhematopoietic cell populations have been identified among the cells shed from diseased organs or tissues. A prominent example is the circulating tumor cells (CTCs) shed from primary tumor sites, which then enter the vascular system and metastasize at other locations [15, 16]. CTCs can represent the molecular characteristics of the originating tumor, and CTC analysis is considered as a noninvasive real-time “liquid biopsy” [17]. In addition to malignant cells, circulating endothelial cells (CECs) are present in patients with benign neoplasms or inflammatory diseases [18–20]. CECs, which normally line blood vessels, are released into the blood because of ongoing endothelial injury in acute myocardial infarction (MI) [21]. Elevated levels of CECs are diagnostic indicators of MI-associated arterial plaque rupture [22]. There have been reports of studies within the past few years identifying CECs as possible biomarkers for primary angiitis [23, 24]. Circulating fetal cells are another clinically important type of circulating cells, as they host the entire fetal genome and provide noninvasive prenatal testing for genetic disorders [25].

In ischemic stroke, the majority of biomarker studies have focused on the proteins and ribonucleic acids and extracellular vesicles that are released from neural cells and cross the blood-brain barrier (BBB) into the peripheral blood after the ischemic brain injury [12, 26–29]. The BBB is a dynamic network that regulates substance exchange between the circulatory system and the brain parenchyma, while also maintaining the homeostasis of the central nervous system (CNS) [30, 31]. Damages to the BBB and cerebral edema formation are significant factors in the occurrence and development of neurological dysfunction in acute and chronic cerebral ischemia [32, 33]. Definitive biomarkers of cerebral ischemia would significantly facilitate the prediction of stroke, especially in the early phase, and complement neuroimaging analyses. However, researchers have yet to identify specific biomarkers available at the cellular level that accurately depict the occurrence and development of ischemic stroke.

Here, we identify NeuN^+^/CD45^−^/DAPI^+^ phenotype nonblood cells (defined as CNCs) in the peripheral blood of mice subjected to middle cerebral artery occlusion (MCAO) and stroke patients. Meanwhile, the enumeration of CNCs in the blood is significantly associated with the severity of brain damage. Our results indicate enumeration of CNCs in circulation may serve as a clinical indicator for the real-time prognosis and progression monitor of the occurrence and development of ischemic stroke and other nervous system disease.

## 2. Results and Discussion

### 2.1. Hypothesis

The BBB protects neurons from the effects of circulating factors and maintains the homeostasis of the central nervous system (CNS), which is necessary for proper synaptic and neuronal functions [5]. Destruction of the BBB is observed in CNS diseases. The BBB is destroyed during the occurrence and development of metastatic tumors in the brain [3] and in neurodegenerative diseases [5]. The BBB in the hippocampus is disrupted in aging humans [7]. The destruction or loss of function of the BBB and the formation of cerebral edema play an important role in the occurrence and development of neurological dysfunction in acute and chronic cerebral ischemia [32, 34, 35].

**Figure 1:**
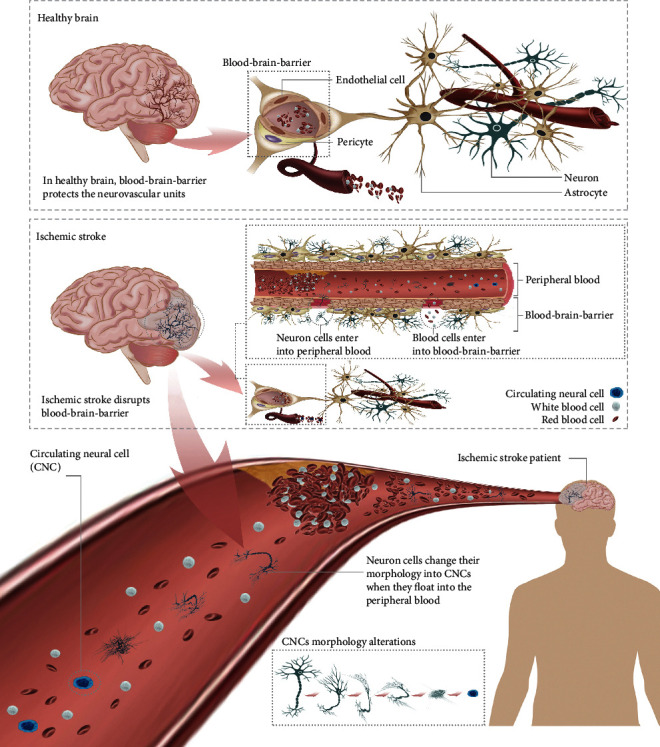
Neural cells are released from the brain and enter the circulatory system when ischemic stroke occurs with blood-brain barrier (BBB) breakdown. The BBB is disrupted when an ischemic brain injury occurs (e.g., ischemic stroke). The injury releases circulating neural cells (CNCs), which shed into the peripheral blood after BBB breakdown. The CNCs gradually lose their original nerve-like morphology.

In a recent study, selective neuron loss was observed in both rodent models and human patients with ischemic stroke [36–40]. Neuronal loss also occurs in neurodegenerative diseases, including Alzheimer's disease [41, 42], Parkinson's disease [43], Huntington's disease [44], amyotrophic lateral sclerosis [45], and multiple sclerosis [46]. We propose that neural cells are released from the brain and enter the circulatory system during the occurrence of ischemic stroke with BBB breakdown (Figure 1). The circulating neural cells (CNCs) would gradually lose their original nerve-like morphology during the escape process. Therefore, we hypothesized that cells with a NeuN^+^/CD45^−^/DAPI^+^ phenotype is present in the peripheral blood of ischemic patients with BBB breakdown. Central nervous system diseases are accompanied by the destruction of the BBB and the loss of neurons, which provides sufficient evidence for our hypothesis. The injury releases circulating neural cells (CNCs), which shed into the peripheral blood when BBB breakdown occurs.

### 2.2. Identification of CNCs in Peripheral Blood from the MCAO Mouse

**Figure 2:**
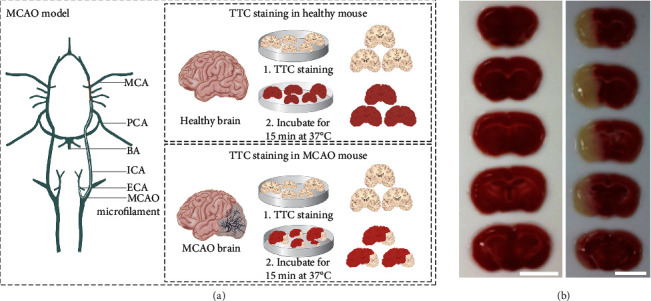
MCAO mouse. (a) The validation workflow of MCAO mice (MCA: middle cerebral artery; PCA: posterior cerebral artery; BA: basilar artery; ICA: internal carotid artery; ECA: external carotid artery). (b) Identification of the cerebral infarction area in the control group (left) and a MCAO mouse 3 days after reperfusion (right) via TTC staining. Scale bar = 5 mm.

**Figure 3:**
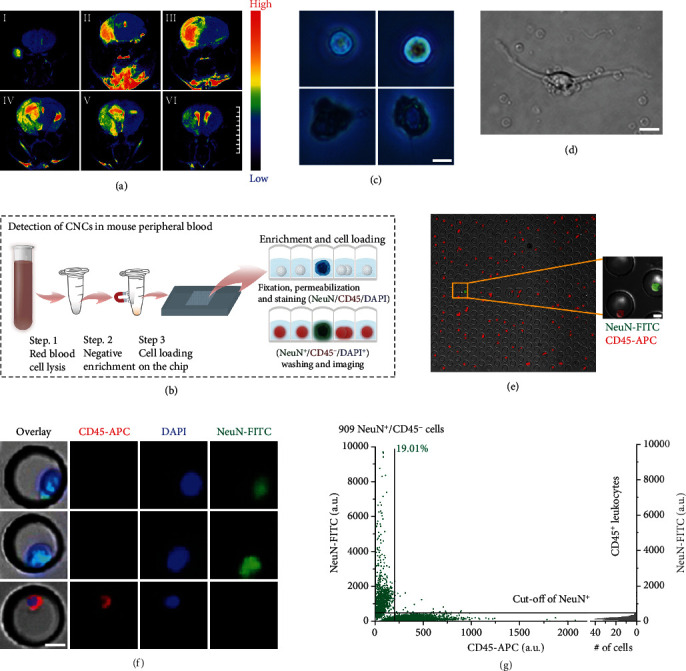
Identification of circulating neural cells in the peripheral blood of mice subjected to middle cerebral artery occlusion (MCAO). (a) Representative MRI of an MCAO mouse. A follow-up T2-weighted scan (I-VI) taken 3 days after reperfusion showed an infarct in the right subcortical area. Scale bar, 10 mm. (b) The workflow of the enrichment CNC detection based on NeuN and CD45 expression. NeuN^+^/CD45^−^/DAPI^+^ cells were identified and termed as CNCs. (c) Bright-field microscopic imaging (40x) of leukocytes (top) and candidate neurons (bottom). Scale bar, 5 *μ*m. (d) The isolated CNCs returned to their original morphology after being cultured in neuronal medium. Scale bar, 5 *μ*m. (e) CNCs collected from MCAO mice that are NeuN^+^/CD45^−^. Scale bar, 10 *μ*m. (f) Representative images of CNCs stained with NeuN-FITC (top and middle panels) and leukocytes expressing CD45-APC (bottom panel). Scale bar, 10 *μ*m. (g) NeuN expression in CD45^−^ and CD45^+^ cell populations in the peripheral blood sample taken from an MCAO mouse. The sample was analyzed on a microwell chip containing a total of 100 blocks. Cells in the 100 blocks were measured and plotted. The gray line is the cut-off value used for the identification of CNCs and was generated via the measurement of CD45^+^ leukocytes. The cut-off value of the fluorescence signal of NeuN corresponds to leukocyte ±3S.D. A total of 909 NeuN^+^/CD45^−^ cells were detected in 0.5 mL of peripheral blood from an MCAO mouse, in which CNCs were defined based on the fluorescent signal values of leukocytes. The number of CNCs represented 19.01% of the total counted cells.

Here, we identified CNCs from MCAO mice with BBB breakdown. We induced cerebral ischemia in mice via 60 min MCAO performed under chloral hydrate at 37°C (Figure 2(a)) [47–49]. Three days after reperfusion, the brain infarct volume of the MCAO mice was recorded and magnetic resonance imaging (MRI) [50, 51] was performed using a clinical imaging system at 3.0 T. The representative cerebral infarction area in the MCAO mice was detected via MRI (Figure 3(a)). Subsequently, the brain was sliced for 2,3,5-triphenyltetrazolium chloride (TTC) staining [52–54], to measure the cerebral infarct in focal ischemia (Figure 2(b)). The successful establishment of this ischemic stroke model was confirmed by the presence of an unstainable cortical infarct area, which remained white (Figure 2(b)).

We identified and enumerated CNCs in MCAO mice using a microwell chip system [55] combined with fluorescence imaging and computational analysis (Figure 3(b) and Figure S1 and S2). In the peripheral blood of the MCAO mice, we found that cells had a rougher surface, lower reflective index, and larger nuclei (Figure 3(c)), which represent typical characteristics of neural cells. The CNCs isolated from peripheral blood samples returned to their neuronal morphology after being cultured in neuronal medium [56–59] (Figure 3(d) and Figure S3 and S4). Both multipotent PDGFR*β*-expressing cells and neuronal outgrowth cells isolated from the peripheral blood of stroke patients can be cultured in vitro [60, 61]. These evidences are essential to prove that circulating neural cells can unexpectedly survive in peripheral blood. Using colocalization visualization, we identified CNCs with a NeuN^+^/CD45^−^/DAPI^+^ phenotype [62–65] (Figures 3(e) and 3(f)). A total of 909 NeuN^+^/CD45^−^ cells were detected in 0.5 mL of peripheral blood from MCAO mice, in which CNCs were defined based on the fluorescent signal values of leukocytes. The number of CNCs represented 19.01% of the total counted cells from the microwell (Figure 3(g) and Figure S5). The 2D scatter plots with more conventional log scaling of intensity are also provided in Figure S6. Except for the NeuN that was already validified in our experiment, MAP2 is also a specific marker of relatively mature neurons [66–69], and Nestin is a marker for neural progenitor cell. Using colocalization visualization, we identified the cells with a MAP2^+^/Nestin^−^/DAPI^+^ phenotype, which was isolated from the peripheral blood of the MCAO mouse model (Figure S7). Then, we identified the cells with MAP2^+^/CD45^−^/DAPI^+^ phenotype in MCAO mice using the microwell chip system combined with fluorescence imaging (Figure S8). Therefore, we defined and cross-verified these rare NeuN^+^/CD45^−^/DAPI^+^ cells as circulating neural cells (CNCs).

### 2.3. Enumeration of CNCs Associates with Brain Damage in the MCAO Mouse Model

**Figure 4:**
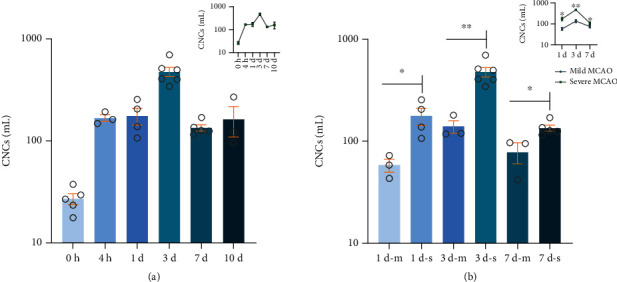
Enumeration of CNCs associates with brain damage in the MCAO mouse model. (a) Number of CNCs per milliliter of peripheral blood of MCAO mice measured at different time points. After the establishment of the MCAO model, the number of CNCs in the peripheral blood of mice reached a peak on the third day after the ischemic brain injury and then began to decrease. The regression curve in the upper right corner is used to reveal the linearity of the data. (b) Within the peripheral blood of mice with different degrees of ischemic brain damage, the number of CNCs detected in the peripheral blood of mice with severe brain injury was higher than that recorded in mice with mild injury. 1 d-m, 3 d-m, and 7 d-m represent mild MCAO mouse groups, while 1 d-s, 3 d-s, and 7 d-s represent severe MCAO mouse groups. The regression curve in the upper right corner is used to reveal the linearity of the data (mild MCAO represents mild MCAO mouse groups, and severe MCAO represents severe MCAO mouse groups). All values are the mean ± SEM. ^∗^*p* < 0.05 and ^∗∗^*p* < 0.01, unpaired *t*-test.

This finding is tantamount to a real-time liquid biopsy of ischemic stroke at the cellular level. The number of CNCs was significantly associated with the severity of the stroke damage in MCAO mice, which represents a blood-based biomarker that can be used to monitor brain injury and disease progression. CNCs in the blood were significantly elevated over the 3 days that followed cerebral ischemic injury in MCAO mice (Figure 4(a), Figure S9 and S10, and Table 1). The regression curve in the upper right corner further demonstrates the linearity of the data. An ischemic stroke typically results from blockage of an artery that supplies blood to the brain, most commonly a branch of one of the internal carotid arteries. As a result, brain cells are deprived of blood. Symptoms of cerebral ischemic generally become worse within the first 2 to 3 days, usually due to swelling caused by excessive fluid (edema) in the brain. In large strokes, the swelling in the brain is typically at its worst stage about 3 days after the stroke begins. Symptoms usually lessen within a few days, as the fluid is absorbed. A regression analysis between CNC variation and brain damage severity revealed that the number of CNCs in the blood was significantly associated with the degree of brain damage (Figure 4(b) and Table 1). Therefore, it is reasonable to observe the enumeration of CNCs drops in the medium and severe models from day 3 to day 7. Meanwhile, the regression curve in the upper right corner further illustrates the enumeration if CNCs is positively correlated with the degree of damage. Our results indicate that CNC variation in the blood reflects the degree of ischemic brain injury and can serve as a potential marker of brain damage. In stroke, BBB breakdown is observed in the venous microvessels and distal capillaries at 0.5-2 h within middle cerebral artery occlusion (MCAO). Most brain cells die if they are deprived of blood for 4.5 hours. Therefore, the time window for stroke diagnosis should be as early as possible.

### 2.4. Identification of CNCs in Peripheral Blood from the Ischemic Stroke Patients

**Figure 5:**
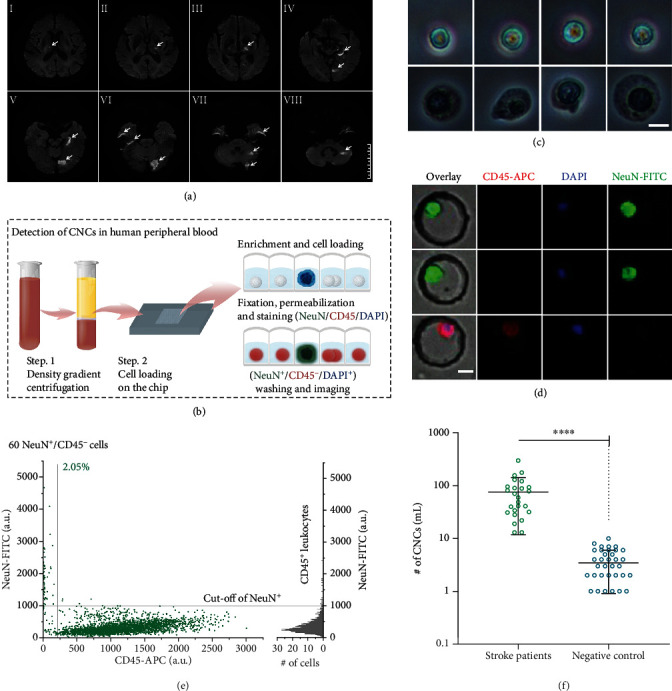
Identification of circulating neural cells in the peripheral blood of patients with ischemic stroke. (a) MRI diffusion-weighted imaging of a representative patient showing areas of new-onset cerebral infarction in the bilateral thalamus, occipital lobe, pons, left hippocampus, and left cerebellar hemisphere. The patient had multiple lacunar infarctions in the bilateral oval, basal ganglia, thalamus, and pons, accompanied by cerebral atrophy (I-VII). Scale bar, 5 cm. (b) Workflow of the detection of the enrichment of neural cells based on NeuN expression and CD45 expression. NeuN^+^/CD45^−^/DAPI^+^ cells were identified as CNCs. (c) Bright-field microscopic imaging (40x) of candidate neural cells (top panel) and leukocytes (bottom panel) in wells after density gradient centrifugation. Scale bar, 5 *μ*m. (d) Representative images of the CNCs identified in peripheral blood samples of patients with stroke. CNCs were stained with NeuN-FITC (top and middle panels), and leukocytes were stained with CD45-APC (bottom panel). Scale bar, 10 *μ*m. (e) NeuN expression in the CD45^−^ and CD45^+^ cell populations in peripheral blood samples of patients with stroke. The sample was analyzed on a microwell chip containing a total of 100 blocks. Cells in the blocks were measured and plotted. The gray line is the cut-off value used for the identification of CNCs and was generated via the measurement of CD45^+^ leukocytes. The cut-off value of the fluorescence signal of NeuN corresponds to leukocyte ±3S.D. A total of 60 NeuN^+^/CD45^−^ cells were detected in the peripheral blood of patients with ischemic stroke, in which candidate cells were defined based on the fluorescent signal intensity of leukocytes. The number of CNCs represented 2.05% of the total counted cells. (f) Statistical analysis showing a significant difference in the number of CNCs detected in 1 mL of peripheral blood between the patient group and the healthy control group. All values are the mean ± SD. ^∗∗∗∗^*p* < 0.0001, unpaired *t*-test.

Next, we used density gradient centrifugation combined with the microwell chip technology to identify CNCs in the blood of 25 patients with stroke (Figure 5). The MRI diffusion-weighted imaging of a representative patient with ischemic stroke identified new-onset cerebral infarctions (Figure 5(a) and Figure S11). The peripheral blood of the patients was processed, examined, and stained (Figures 5(b)–5(d)), and the presence of CNCs was confirmed in the patients. The CNCs isolated from patients with ischemic stroke had a NeuN^+^/CD45^−^/DAPI^+^ phenotype (Figure 5(d) and Figure S12 and S13). The scatter plot reports the NeuN and CD45 fluorescence intensity of all enriched nucleated cells in peripheral blood samples from patients with ischemic stroke (Figure 5(e)). The cut-off value used for the identification of CNCs was generated via the measurement of CD45^+^ leukocytes. Under the cut-off, a total of 60 NeuN^+^/CD45^−^/DAPI^+^ cells were detected. The number of CNCs represented 2.05% of the total counted cells on the microwell. (Figure 5(e)). The 2D scatter plots with more conventional log scaling of intensity are also provided in Figure S14. The number of CNCs in the peripheral blood of negative controls was significantly lower than that detected in the patients with stroke (Table 2, Table S1 and S2, and Figures S15-S17). Statistical analysis showed a significant increase in the number of CNCs detected in 1 mL of peripheral blood between the negative control group and the stroke patient group (*p* < 0.0001) (Figure 5(f) and Table S1).

We have performed further experiments by immunofluorescence staining glucose metabolism of 2-NBDG [70] and NeuN simultaneously on the cells which were isolated from the peripheral blood of the ischemic stroke patient. 2-NBDG (2-(N-(7-nitrobenz-2-oxa-1,3-diazol-4-yl) amino)-2-deoxyglucose) is an indicator to monitor glucose uptake in live cells and commonly perceived as an indicator of cell viability [71, 72]. We performed 2-NBDG/CD45 costaining, followed by on-chip cell fixation, permeabilization, and sequential staining with NeuN/CD45/DAPI again. Therefore, we can use the ratio between NeuN^+^/CD45^−^/DAPI^+^/2-NBDG^+^ (live CNCs) and NeuN^+^/CD45^−^/DAPI^+^ (total CNCs) to evaluate the survival rate of CNCs in peripheral blood. The patient blood draw was done between 20 and 24 hours after hospitalization and quickly fixed for analysis (of note, the patients have already developed stroke symptoms before sending to hospital; therefore, the CNCs in peripheral blood were indeed longer than 24 hours before acquisition). Our data indicate that this ratio is 60.4% for our patient cohort. Therefore, at least >60% CNCs in peripheral blood can survive after 1 day. Notably, since ischemic stroke is a continuous process, the release of CNC is also a continuous state (this process is similar to the release process of CTC in tumor patients). Therefore, this survival evaluation is an overall statistic from the occurrence of stroke to the blood draw, which may not exactly represent the CNCs released into blood at time point zero (Figure S18).

## 3. Conclusion

In summary, this study indicated that neural cells spread from the brain-injury area to the peripheral blood during BBB breakdown. We identified these neural cells for the first time and defined them as circulating neural cells (CNCs). The number of CNCs was associated with brain damage. The identified CNCs may serve as a biomarker for the analysis of ischemic stroke development. Our findings may ultimately support the development of an assay to predict the imminent risk of ischemic stroke and other nervous system diseases.

## 4. Materials and Methods

### 4.1. Mice

The animal experiments included in this study were approved by the Institutional Animal Care and Use Committee of the Institute for Personalized Medicine, School of Biomedical Engineering, Shanghai Jiao Tong University, and were performed in accordance with regulations and guidelines. The Biomedical Research Project ethics review approval number is 2018043. Eight-week-old male C57BL/6 mice weighing 24-28 g at the time of surgery were purchased from Shanghai Jiesijie Experimental Animal Co. Ltd. and used for all experiments in this study. The mice were housed at five animals per cage and maintained on a 12 h/12 h light/dark cycle with ad libitum access to water and rodent chow.

### 4.2. Middle Cerebral Artery Occlusion (MCAO) Model

Transient cerebral ischemia in mice was induced based on the literature [48, 52]. The mice were anesthetized via intraperitoneal injection of chloral hydrate (400 mg/kg). After disinfecting with iodophor, the midline neck was incised to dissect the right common carotid artery (CCA). The CCA was temporarily occluded by a 3-0 silk suture. A permanent suture was placed around the external carotid artery (ECA), and another temporary suture was placed on the ECA distal to the bifurcation. The left internal carotid artery (ICA) was clipped using a hemostatic forceps clamp, to avoid bleeding. After cutting a small hole into the ECA between the permanent and temporary sutures, a 30 mm long silicon-coated (about 5 mm was coated with silicon) monofilament suture was introduced into the ECA and then inverted into the ICA. The suture was tightly tied around the monofilament, to prevent bleeding, and the reverse-action tweezers were removed. The MCAO monofilament was introduced to occlude the origin of the MCA in the circle of Willis (9-10 mm insertion beyond the bifurcation of the ECA and CCA). The suture on the ECA was tightly tied to fixate the monofilament. The temporary suture was removed from the CCA. After 60 min of occlusion, the monofilament suture was withdrawn to allow reperfusion.

### 4.3. Magnetic Resonance Imaging (MRI)

MCAO mice were anesthetized via intraperitoneal injection of chloral hydrate (400 mg/kg) and fixed to a mouse cradle. Magnetic resonance imaging was performed using a clinical imaging system at 3.0 T. Coronal T1W and T2W_TSE sequences were obtained using a spin-echo technique (TR = 3842.64 ms and TE = 27.15 ms for the T1W images; TR = 3430.49 ms and TE = 105.48 ms for the T2W images) [50, 51]. Other imaging parameters included a 1 mm slice thickness.

### 4.4. Triphenyltetrazolium Chloride (TTC) Staining

The mice were euthanized at different time points after stroke. TTC staining was carried out as described previously [51–53]. The brains were quickly removed and chilled at −80°C for 4 min, to slightly harden the tissue. Five or six coronal brain sections (thickness, 1 mm) were prepared from the olfactory bulb to the cerebellum and then stained with 2% TTC (Sigma) for 15 min at 37°C.

### 4.5. Candidate Circulating Neural Cell Culture

Candidate CNCs were isolated from peripheral blood samples from MCAO mice and plated on poly-l-ornithine (PLO; 15 *μ*g/ml; Sigma-Aldrich)/laminin (1 *μ*g/ml; Sigma-Aldrich)-coated 96-well plates in neuronal medium containing B27 Plus Supplement (2%; Gibco), glutamine (1X; Gibco), and Neurobasal Plus (Gibco). One day after plating, glutamine was withdrawn from the medium and candidate CNCs were incubated for 3-7 days.

### 4.6. Clinical Samples

This study was approved by the Ethical Review Board of Wenling Hospital Affiliated to Wenzhou Medical University. The ethical approval number is KY-2019-036. The Chinese clinical trial registration number is ChiCTR2000034666. From November 2019 through to June 2020, a cohort of 25 validated stroke samples and 38 normal control samples that met the acceptance criteria was measured and recorded. Patients admitted to the hospital were chosen and measured on the second or third day of treatment. Two EDTA anticoagulant tubes (BD Vacutainer) of venous blood with a minimum of 3 ml were collected and delivered to the laboratory within 5 h. Clinical information was collected from the patient records.

### 4.7. Microwell Chip

The microwell chip was fabricated in poly(dimethylsiloxane) (PDMS) using standard microfabrication soft-lithographic techniques [73, 74]. A replicate for molding the PDMS was obtained by patterning a silicon wafer using photoresist SU-8 2050. The PDMS prepolymer (Sylgard 184) was mixed at a ratio of 10 : 1 and subsequently cast on a lithographically patterned replicate. After curing at 80°C for 2 h, the PDMS component was separated from the replicate. The microwell chip was attached to a clean glass slide, hydrophilized by plasma, and blocked with 3% BSA. The chip contained 112,000 microwells with a diameter and depth of 30 and 20 *μ*m, respectively.

### 4.8. Peripheral Blood Collection and Enrichment of Circulating Neural Cells

Peripheral blood samples of the MCAO and control mice were collected from their hepatic portal vein using EDTA anticoagulant tubes. Subsequently, 5 mL of red blood cell lysing buffer (BD) was added to the tubes, to lyse the red blood cells for 15 min. After centrifuging at 300 × g for 5 min, the nucleated cell pellet was resuspended and washed with MojoSort Buffer, followed by centrifugation at 300 × g for 5 min (the supernatant was discarded). For negative separation of candidate CNCs, 2 *μ*L of anti-CD45-coated nanobeads was added to the cell suspension and incubated on ice for 15 min, followed by the addition of 0.5 mL of MojoSort Buffer and thorough mixing. The tube was then placed in a magnetic separator for 5 min, and the clear solution containing the candidate CNCs was collected for subsequent characterization.

All blood samples from patients with stroke and healthy donors were collected in EDTA anticoagulant tubes and delivered to a central laboratory within 2 h at 4°C. For the enrichment of target cells via density gradient centrifugation, 5 ml of blood sample was first mixed with 75 *μ*l of CTC enrichment antibody cocktail (RosetteSep CTC Enrichment Cocktail Containing Anti-CD36, StemCell) and incubated at room temperature for 20 min. Next, 15 mL of HBSS with 2% FBS (Gibco) was added and thoroughly mixed. The mixture was carefully added along the wall of a Sepmate tube (SepMate-50 (RUO), StemCell), followed by the addition of 15 mL of density gradient liquid (Lymphoprep, Stemcell) to the tube through the middle hole. After centrifugation at 1200 × g for 20 min, the top layer of the supernatant (~10 mL) was removed and the supernatant (~10 mL) that remained above the barrier of the Sepmate tube was poured out into a new centrifuge tube. After centrifugation at 600 × g for 8 min, the supernatant was removed and 0.5 mL of red blood cell lysing buffer (BD Biosciences) was added, to lyse the red blood cells for 5 min at room temperature. After centrifugation at 300 × g for 5 min at 4°C, ~400 *μ*l of supernatant was removed and the cell pellets were resuspended in the remaining ~100 *μ*l of supernatant, followed by application onto the 3% BSA (Sigma)-treated microwell chip.

### 4.9. Immunofluorescence Staining

Cell suspension was applied onto the 3% BSA (Sigma)-treated microwell chip as a monolayer and wait for 5 min until cells sitting down in the 200,000 microwells, leading to 1~2 cells per well in average. All cells on the chip were briefly deprived of glucose for 10 min by applying 400 *μ*l of glucose free DMEM and then exposed to 0.4 mM 2-NBDG for 15 min in a cell incubator. After extensive washing with the cold PBS, an ImageXpress Micro XLS Widefield High Content Screening System (Molecular Devices) scanned the chip and imaged all cells in the microwells. After cell fixation (4% PFA, 30 min) and permeabilization (0.3% Triton X-100, 15 min), blocking solution consisting of 3% BSA and 10% Normal Goat Serum was applied onto the chip for 1 h of blocking, followed by incubation with anti-NeuN primary antibody anti-RBFOX3 (Sigma, 1 : 1000) overnight at 4°C and subsequent incubation with FITC-conjugated secondary antibody (Invitrogen) and APC-conjugated anti-CD45 (BD) and DAPI (Beyotime Biotechnology) for 2 h at the room temperature. After extensive washing with PBS, an ImageXpress Micro XLS Widefield High Content Screening System (Molecular Devices) scanned the chip and imaged all cells in the microwells in three fluorescent colors (CD45: CY5 and NeuN: FITC and DAPI) as well as the bright field. The antibodies used in the mouse experiment are recombinant anti-NeuN antibody (Abcam, 1 : 1000), APC anti-mouse CD45 (Biolegend), chicken anti-MAP2 (Abcam, 1 : 1000), and anti-Nestin (Millipore). After the immunofluorescence staining is finished, use the Image J software to read the fluorescence signal value of the cells.

### 4.10. Statistical Methods

Statistical analyses were performed using GraphPad PRISM 8. The statistical significance of the differences between two groups was assessed using two-tailed Student's *t*-test using *p* < 0.05 as the significance threshold.

## Figures and Tables

**Table 1:** The number of CNCs in the peripheral blood of mice at different reperfusion time.

Reperfusion time	Case #	Volume/blood mL	NeuN^+^/CD45^−^ cells	Damage severity	CNCs/mL
0 h^a)^	1	0.82	22	/^b)^	26.8
	2	0.74	13	/	17.6
	3	0.4	15	/	37.5
	4	0.3	7	/	23.3
	5	0.88	26	/	29.5

4 h	6	0.35	52	++	148.5
	7	0.6	116	++	193.3
	8	0.4	65	++	162.5

1 d^a)^	9	0.3	13	+	43.3
	10	0.75	44	+^b)^	58.6
	11	0.8	58	++	72.5
	12	0.43	46	++	107
	13	0.5	69	++	138
	14	0.5	103	++	206
	15	0.55	141	++	256

3 d	16	0.5	59	+	118
	17	0.6	72	+	120
	18	0.55	99	+	180
	19	0.7	286	++	408.6
	20	0.6	207	++	345
	21	0.6	299	++	498.3
	22	0.41	166	++	404
	23	0.5	257	++	514
	24	0.5	350	++	700

7 d	25	0.5	21	+	42
	26	0.64	61	+	95
	27	0.64	62	+	97
	28	0.6	69	++	115
	29	0.6	75	++	125
	30	0.62	79	++	127
	31	0.64	87	++	136
	32	0.1	17	++	170

10 d	33	0.75	72	+	96
	34	0.7	86	+	122.8
	35	0.3	81	++	270

^a)^Abbreviations: h: hour; d: day; ^b)^+, exhibition of symptoms, the number of which represents severity; /, symptomless.

**Table 2:** Clinic-pathological characteristics and CNC enumeration of clinical specimens^a)^.

Subjects	*n*	Age (range) (years)	% male	MRI	NIHSS (range)	CNCs/mL (low, high)
Ischemic stroke	25	41-89	76%	+^b)^	1-33	76.36 (13-299)
Negative control	38	27-81	47.36%	/^b)^	/	3.45 (0-10)

^a)^Detailed patience information spread sheet is available as supplementary file Table S1. ^b)^+, symptoms; /, symptomless.

## Data Availability

All data needed in the paper are present in the paper and in the Supplementary section. Additional data related to this paper may be requested from the authors.

## Supplementary Materials

Supplementary Materials Figure S1: images of the microwell chip. Figure S2: computerized high-speed fluorescent microscope scans of the microwell chip containing 400 blocks. Figure S3: representative morphologies of CNCs isolated from peripheral blood samples of MCAO mice. Figure S4: immunofluorescence results of a CNC that has reverted to a neuron morphology. Figure S5: NeuN expression of CD45^−^ and CD45^+^ cell populations in a peripheral blood sample taken of MCAO mouse. Figure S6: the plot 2D scatter plots of NeuN expression of CD45^−^ and CD45^+^ cell populations in a peripheral blood sample taken of MCAO mouse. Figure S7: representative images of the cells with MAP2^+^/Nestin^−^/DAPI^+^ phenotype identified in peripheral blood sample of MCAO mouse model. Figure S8: representative images of the cells with MAP2^+^/CD45^−^/DAPI^+^ phenotype of MCAO mice. Scale bar = 10 *μ*m. Figure S9: the TTC staining results of mouse brain tissue at different reperfusion time points. Figure S10: representative images of CNCs of MCAO mice. Figure S11: the representative MRI images from different degrees of cerebral infarction. Figure S12: bright field and fluorescent images of CNCs and leukocytes of stroke patients. Figure S13: representative images of CNCs identified in the peripheral blood sample of stroke patients. Figure S14: the plot 2D scatter plots of NeuN expression of CD45^−^ and CD45^+^ cell populations in a peripheral blood sample taken of human patient. Figure S15: the representative MRI images from different degrees of cerebral infarction Figure S16: the proportion of the cerebral infarction area in MRI images. Figure S17: statistical correlation between CNCs enumeration and extent of stroke between mild group and intermediate group patients. Figure S18: representative images of cells with NeuN^+^/CD45^−^/DAPI^+^/2-NBDG^+^ phenotype identified in peripheral blood sample of ischemic stroke patient. Table S1: pathological characteristics and CNC enumeration of clinical samples. Table S2: clinical and pathological data of the stroke patients.
