# Supplementary material for: Single-Cell Microwell Platform Reveals Circulating Neural Cells as a Clinical Indicator for Patients with Blood-Brain Barrier Breakdown
Source: Research (Wash D C). 2021 Jul 8;2021:9873545. doi: 10.34133/2021/9873545 (PMC8285994; doi:10.34133/2021/9873545)
Supplement: Supplementary Materials — Figure S1: images of the microwell chip. Figure S2: computerized high-speed fluorescent microscope scans of the microwell chip containing 400 blocks. Figure S3: representative morphologies of CNCs isolated from peripheral blood samples of MCAO mice. Figure S4: immunofluorescence results of a CNC that has reverted to a neuron morphology. Figure S5: NeuN expression of CD45− and CD45+ cell populations in a peripheral blood sample taken of MCAO mouse. Figure S6: the plot 2D scatter plots of NeuN expression of CD45− and CD45+ cell populations in a peripheral blood sample taken of MCAO mouse. Figure S7: representative images of the cells with MAP2+/Nestin−/DAPI+ phenotype identified in peripheral blood sample of MCAO mouse model. Figure S8: representative images of the cells with MAP2+/CD45−/DAPI+ phenotype of MCAO mice. Scale bar = 10 μm. Figure S9: the TTC staining results of mouse brain tissue at different reperfusion time points. Figure S10: representative images of CNCs of MCAO mice. Figure S11: the representative MRI images from different degrees of cerebral infarction. Figure S12: bright field and fluorescent images of CNCs and leukocytes of stroke patients. Figure S13: representative images of CNCs identified in the peripheral blood sample of stroke patients. Figure S14: the plot 2D scatter plots of NeuN expression of CD45− and CD45+ cell populations in a peripheral blood sample taken of human patient. Figure S15: the representative MRI images from different degrees of cerebral infarction Figure S16: the proportion of the cerebral infarction area in MRI images. Figure S17: statistical correlation between CNCs enumeration and extent of stroke between mild group and intermediate group patients. Figure S18: representative images of cells with NeuN+/CD45−/DAPI+/2-NBDG+ phenotype identified in peripheral blood sample of ischemic stroke patient. Table S1: pathological characteristics and CNC enumeration of clinical samples. Table S2: clinical and pathological data of th [file 9873545.f1.docx]

**Supplementary Information**

**Single-cell Microwell Platform Reveals Circulating Neural Cells as a Clinical Indicator for Patients with Blood−Brain Barrier Breakdown**

Yu Zhang^1^, Antony R. Warden^1^, Khan Zara Ahmad^1^, Yanlei Liu^2^, Xijun He^3^, Minqiao Zheng^4^, Xinlong Huo^5^, Xiao Zhi^1^, Yuqing Ke^1^, Hongxia Li^1^, Sijia Yan^1^, Wenqiong Su^1^, Deng Cai^6^, Xianting Ding^1^*

^1^State Key Laboratory of Oncogenes and Related Genes, Institute for Personalized Medicine, School of Biomedical Engineering, Shanghai Jiao Tong University, 1954 Huanshan Road, Shanghai 200030, P.R. China.

^2^Shanghai Engineering Research Centre for Intelligent Diagnosis and Treatment Instrument, Department of Instrument Science and Engineering, School of Electronic Information and Electrical Engineering, Shanghai Jiao Tong University, 800 Dongchuan Road, Shanghai 200240, P.R. China.

^3^Department of Neurosurgery, Wenling Hospital Affiliated to Wenzhou Medical University, Chuan’an Nan Road, Chengxi Subdistrict, Wenling 317500, Zhejiang, P.R. China.

^4^Central Laboratory, Wenling Hospital Affiliated to Wenzhou Medical University, Chuan’an Nan Road, Chengxi Subdistrict, Wenling 317500, Zhejiang, P.R. China.

^5^Department of Neurology, Wenling Hospital Affiliated to Wenzhou Medical University, Chuan’an Nan Road, Chengxi Subdistrict, Wenling 317500, Zhejiang, P.R. China.

^6^Department of Thoracic Surgery, Shanghai Chest Hospital, Shanghai Jiao Tong University, 241 West Huaihai Road, Shanghai 200030, China.

Correspondence should be addressed to Xianting Ding; dingxianting@sjtu.edu.cn


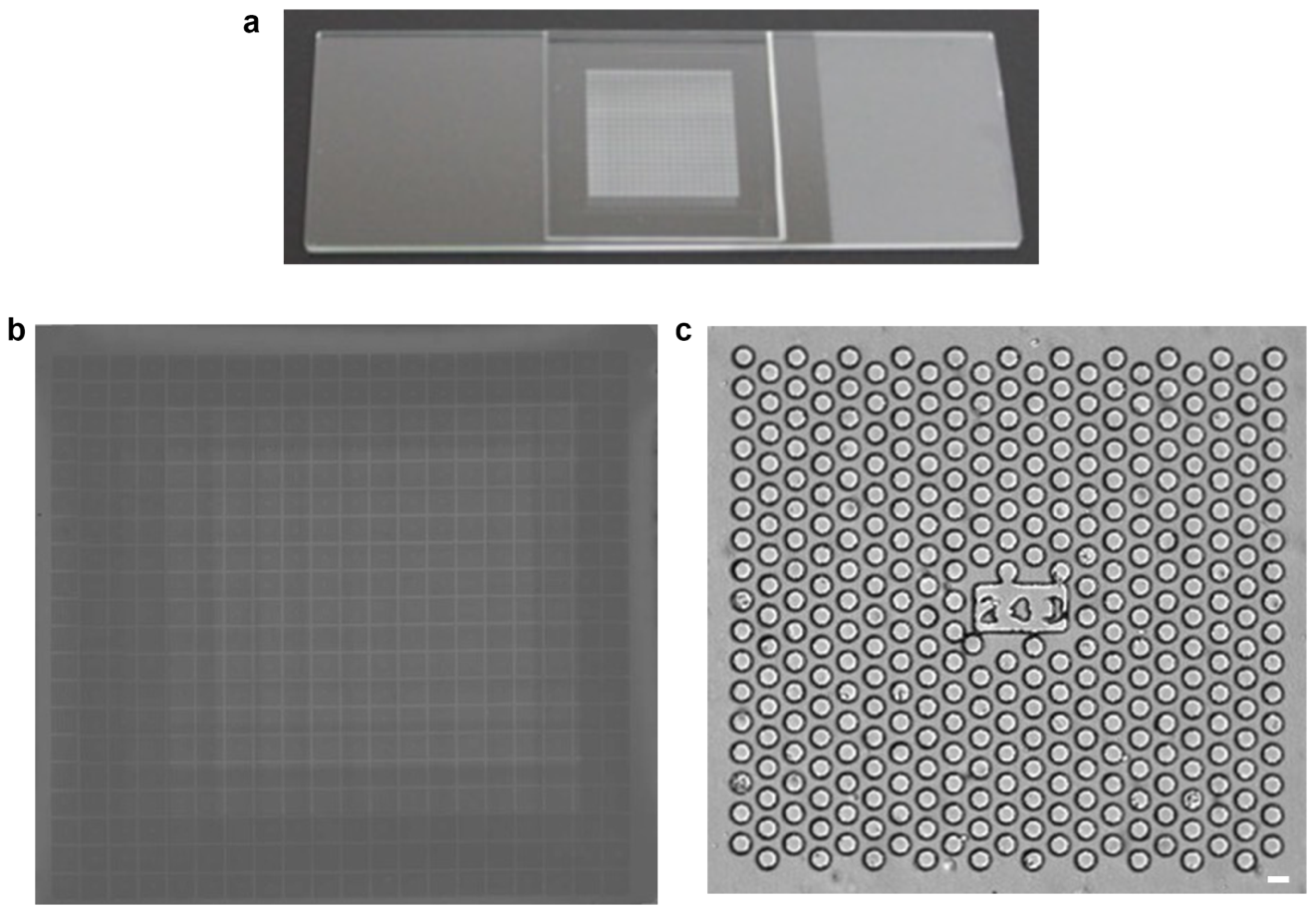


**Figure S1. Images of the microwell chip. a**. This chip has 112,000 microwells with a diameter and height of 30 μm by 20 μm. **b**. A computerized high-speed fluorescent microscope scanned the whole microwell chip containing 400 blocks. **c**. A magnification of one of the blocks in (b). Scale bar = 30 μm.


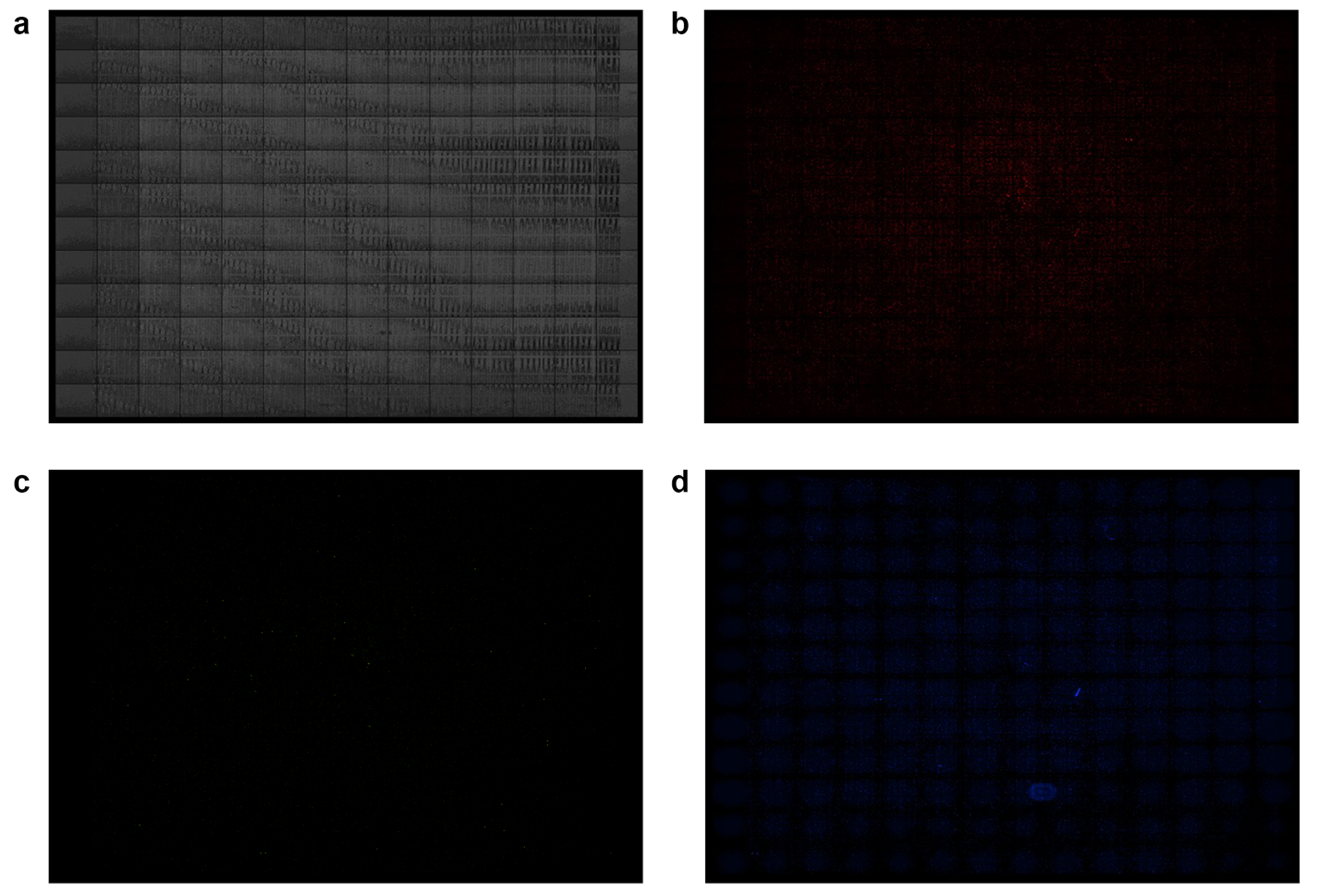


**Figure S2. Computerized high-speed fluorescent microscope scans of the microwell chip containing 400 blocks. a**. The bright field channel. **b**. The CD45-APC channel. **c**. The NeuN-FITC channel. **d**. The DAPI channel.

**
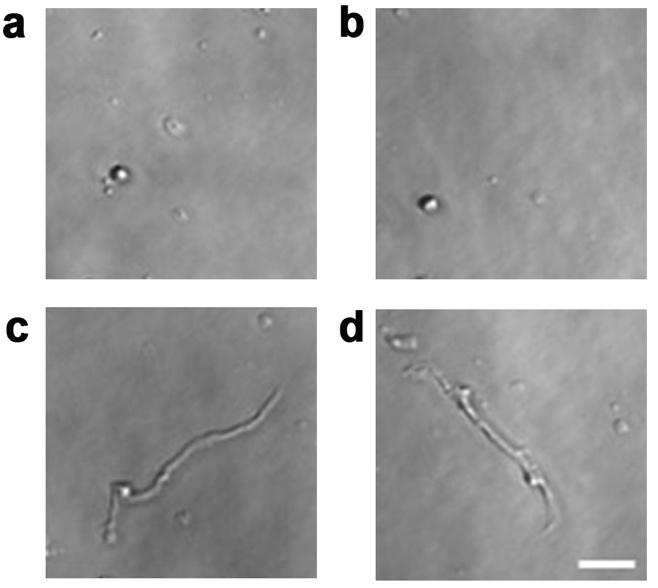
**

**Figure S3. Representative morphologies of CNCs isolated from peripheral blood samples of MCAO mice.** (**a-b**) CNCs isolated from peripheral blood of MCAO mice. (**c-d**) Representative CNCs that reverted to a neuron morphology after being cultured in neuronal-medium. Scale bar = 10 μm.

**
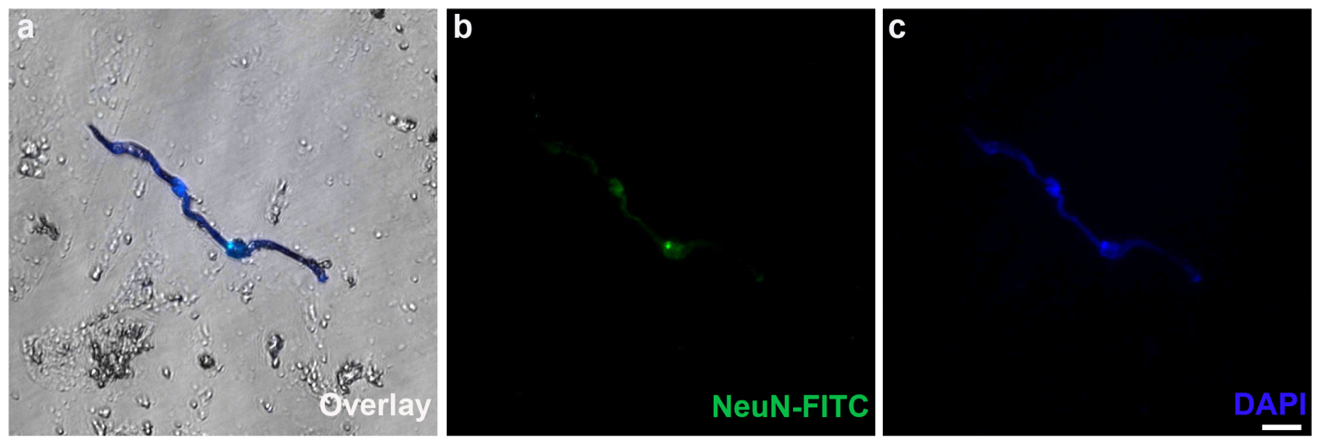
**

**Figure S4. Immunofluorescence results of a CNC that has reverted to a neuron morphology.** (**a-c**) Fluorescence results show NeuN (neuronal classic marker) positive and DAPI positive. Scale bar = 10 μm.

**
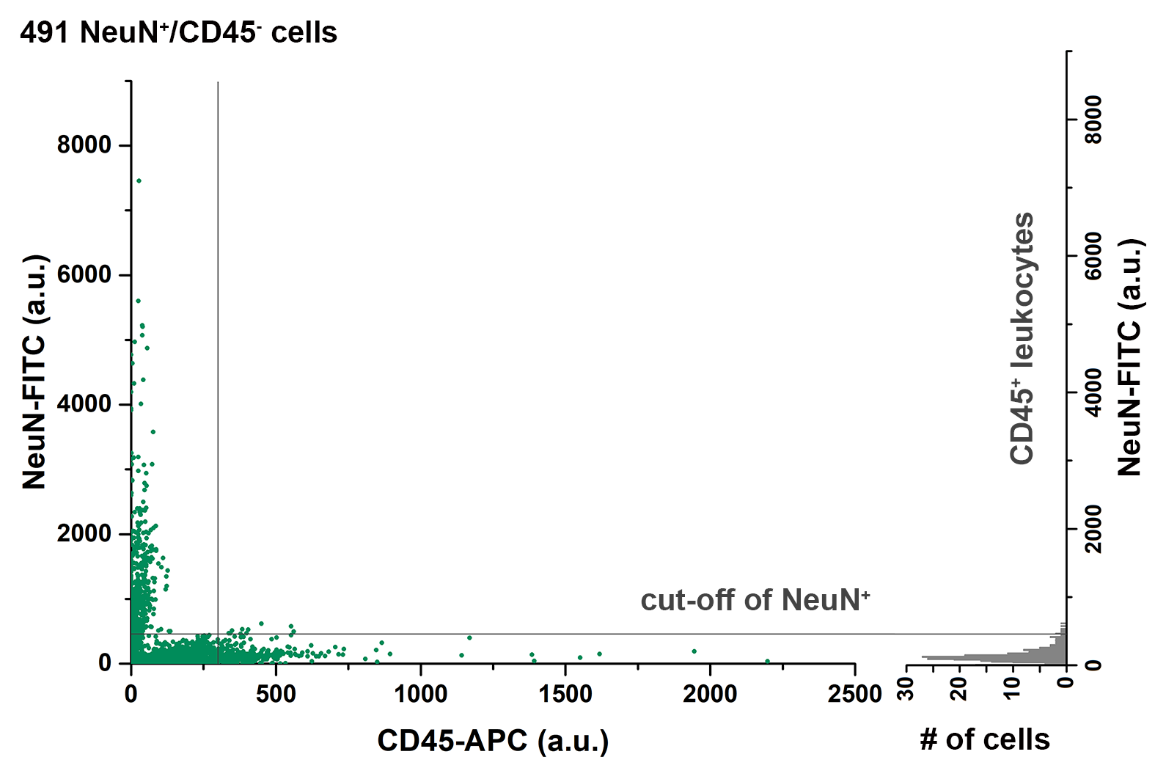
**

**Figure S5. NeuN expression of CD45− and CD45+ cell populations in a peripheral blood sample taken of MCAO mouse.** The sample was analyzed on a microwell chip containing a total of 100 blocks. Cells within the 100 blocks were measured and plotted. A total of 491 NeuN+/CD45− cells were detected in the peripheral blood of MCAO mouse in which candidate cells were defined based on the fluorescent signal values of leukocytes.


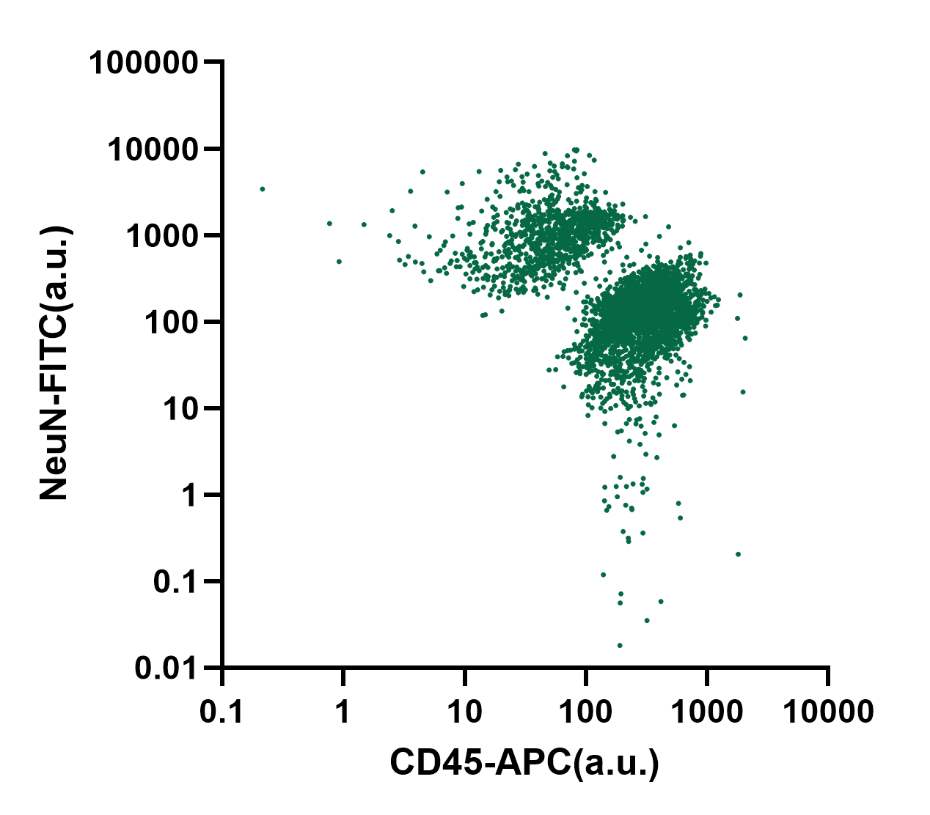


**Figure S6.** The plot 2D scatter plots of NeuN expression of CD45− and CD45+ cell populations in a peripheral blood sample taken of MCAO mouse.


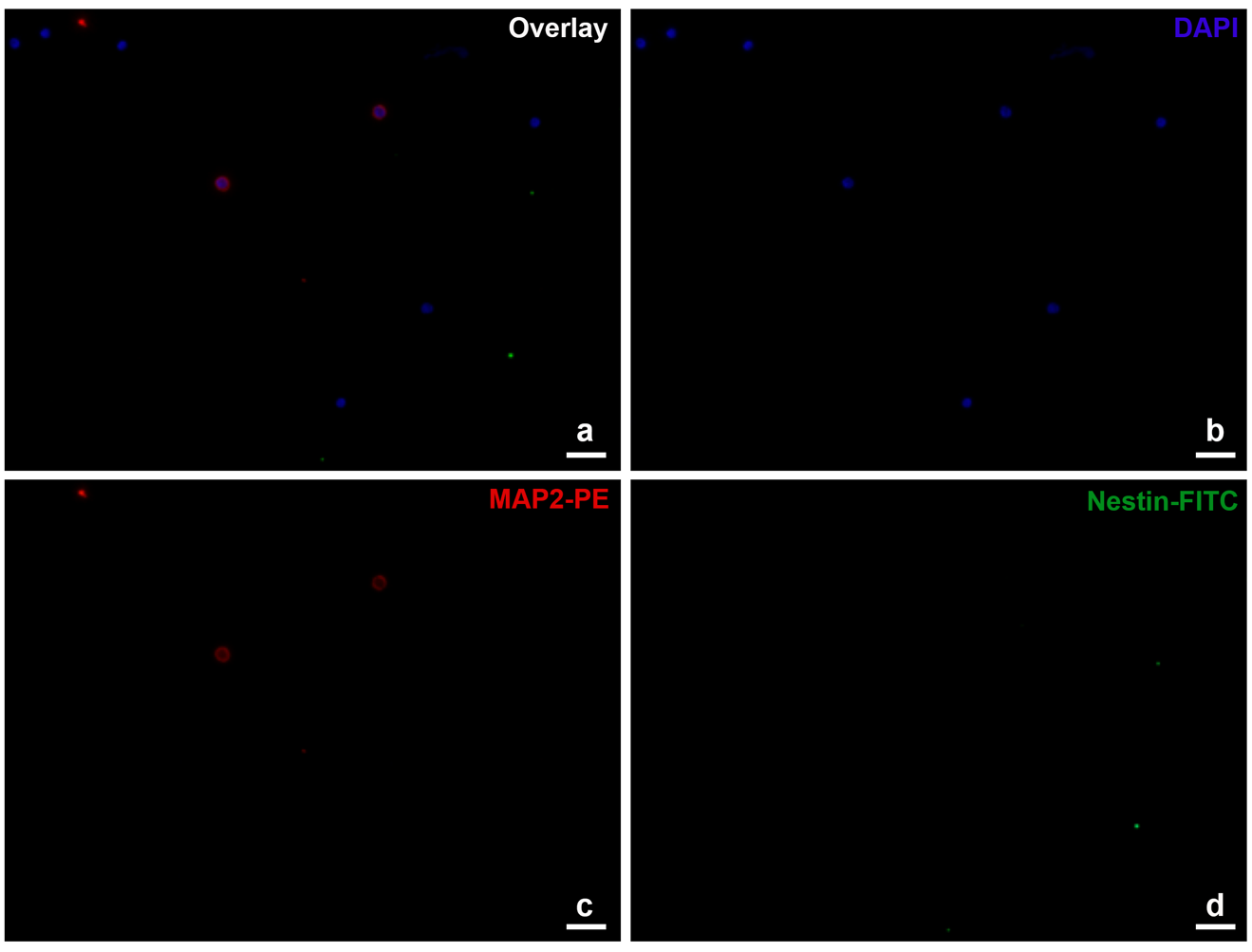


**Figure S7. Representative images of the cells with MAP2+/Nestin-/DAPI+ phenotype identified in peripheral blood sample of MCAO mouse model. a**. Fluorescence results show MAP2 positive and DAPI positive. **b**. The DAPI channel. **c**. The MAP2-PE channel. **d**. The Nestin-FITC channel. Scale bar = 20 μm.


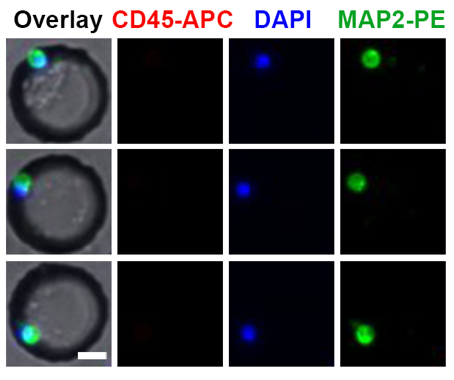


**Figure S8.** Representative images of the cells with MAP2^+^/CD45^-^/DAPI^+^ phenotype of MCAO mice. Scale bar = 10 μm.

**
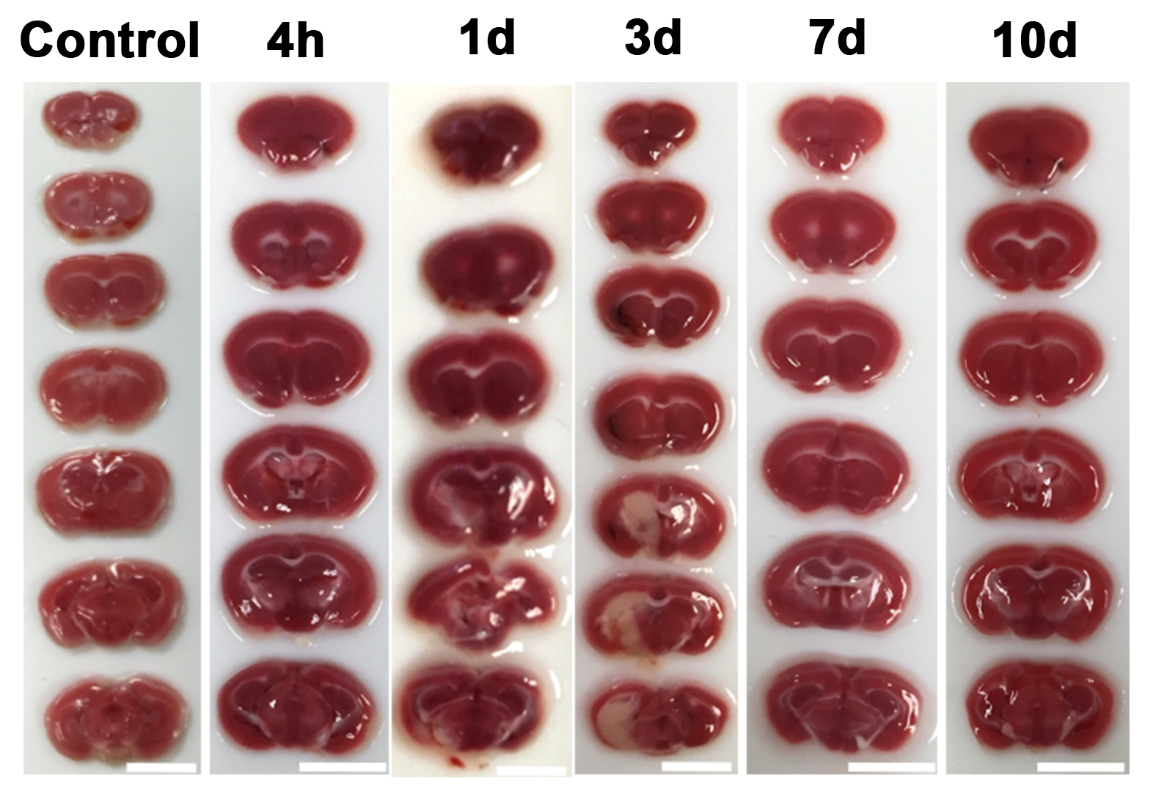
**

**Figure S9. The TTC staining results of mouse brain tissue at different reperfusion time points.** Control group without ischemic brain injury; h, hour; d, day. Scale bar = 5 mm.

**
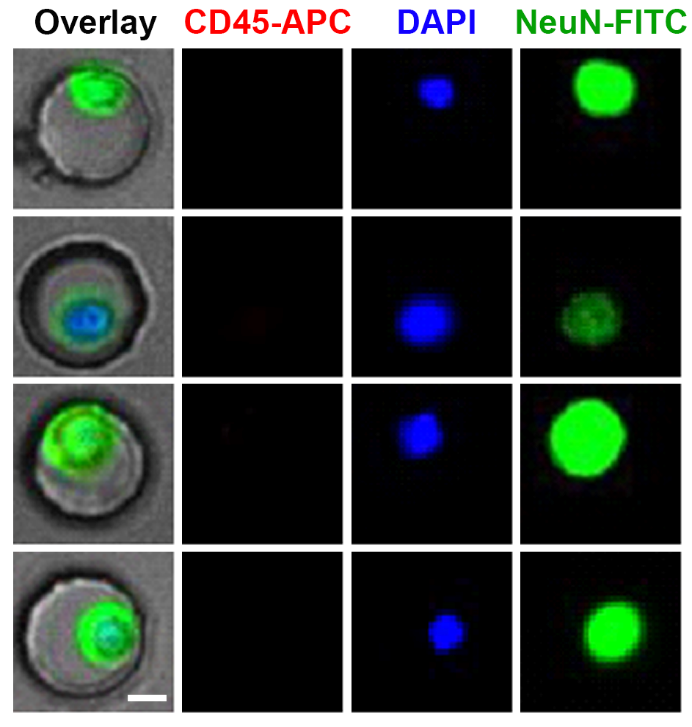
**

**Figure S10. Representative images of CNCs of MCAO mice.** The CNCs were stained with NeuN-FITC and DAPI. Scale bar = 10 μm.


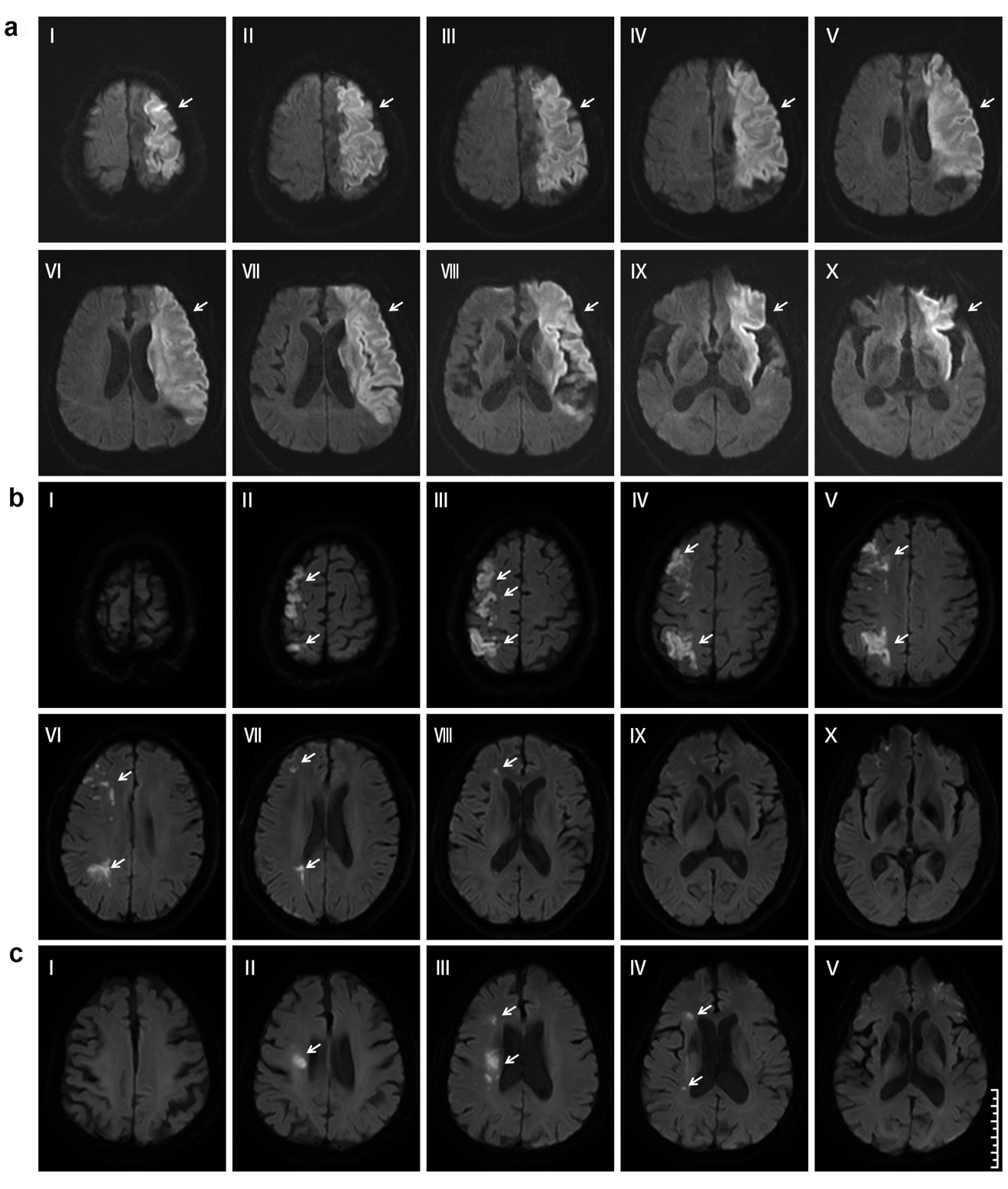


**Figure S11. The representative MRI images from different degrees of cerebral infarction. a.** MRI diffusion-weighted imaging of a representative patient from severe group showed a new cerebral infarct in the left parietal frontal lobe. The patient had multiple lacunar infarctions in the bilateral semi-oval area, basal ganglia and pons, accompanied by brain atrophy (Ⅰ−Ⅹ). **b.** MRI diffusion-weighted imaging of a representative patient from intermediate group showed new cerebral infarction in the right frontal and parietal lobe. The patient had multiple lacunar infarctions in the bilateral semi-oval area, basal ganglia, and pons, accompanied by brain atrophy(Ⅰ−Ⅹ). **c.** MRI diffusion-weighted imaging of a representative patient from mild group showed new cerebral infarction in the right semi-oval area. Scale bar, 5 cm(Ⅰ−Ⅴ).

**
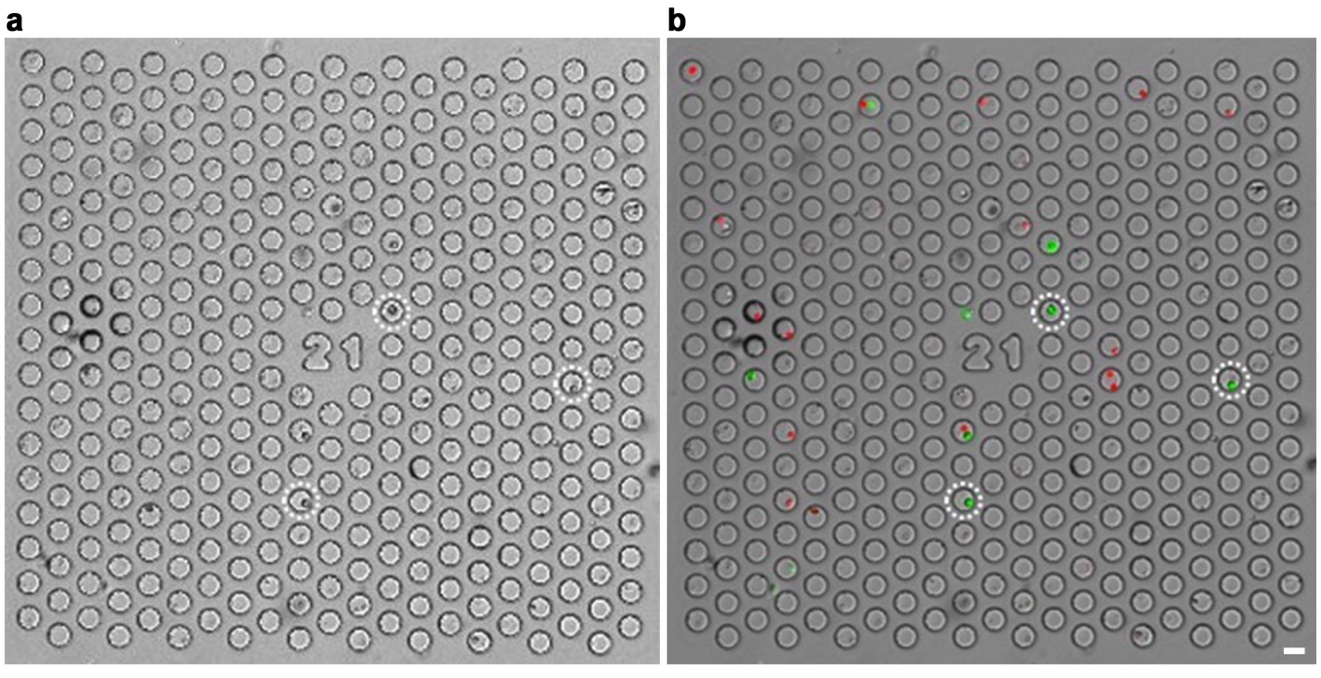
**

**Figure S12. Bright field and fluorescent images of CNCs and leukocytes of stroke patients. a**. This figure represents block 21 on the chip in which cells are sitting in the microwells. Most microwells contained no cells and only a few microwells contained a single cell. The bright field of the microwell chip with the CNCs and leukocytes. **b**. The fluorescent images of the CNCs and leukocytes sitting in the bottom of the microwell chip after treated with NeuN-FITC and CD45-APC, which are shown as green and red, respectively. CNCs of the patients are NeuN+/CD45−. Scale bar = 30 μm.

**
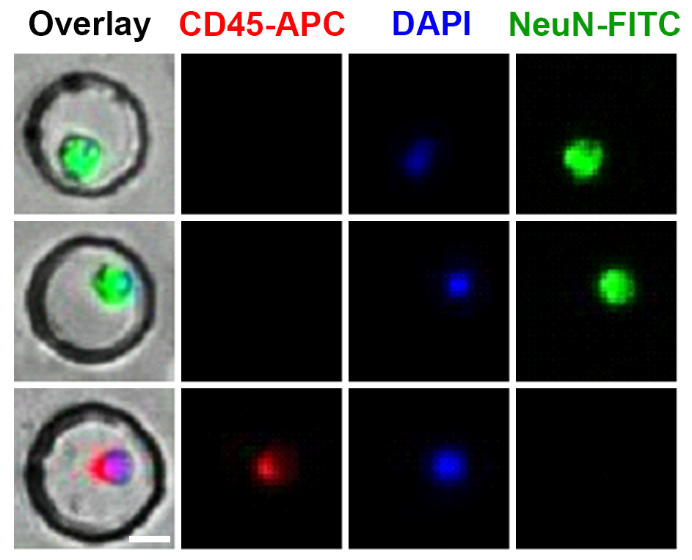
**

**Figure S13. Representative images of CNCs identified in the peripheral blood sample of stroke patients.** The CNCs were stained with NeuN-FITC and the leukocytes were stained with CD45-APC. Scale bar = 10 μm.


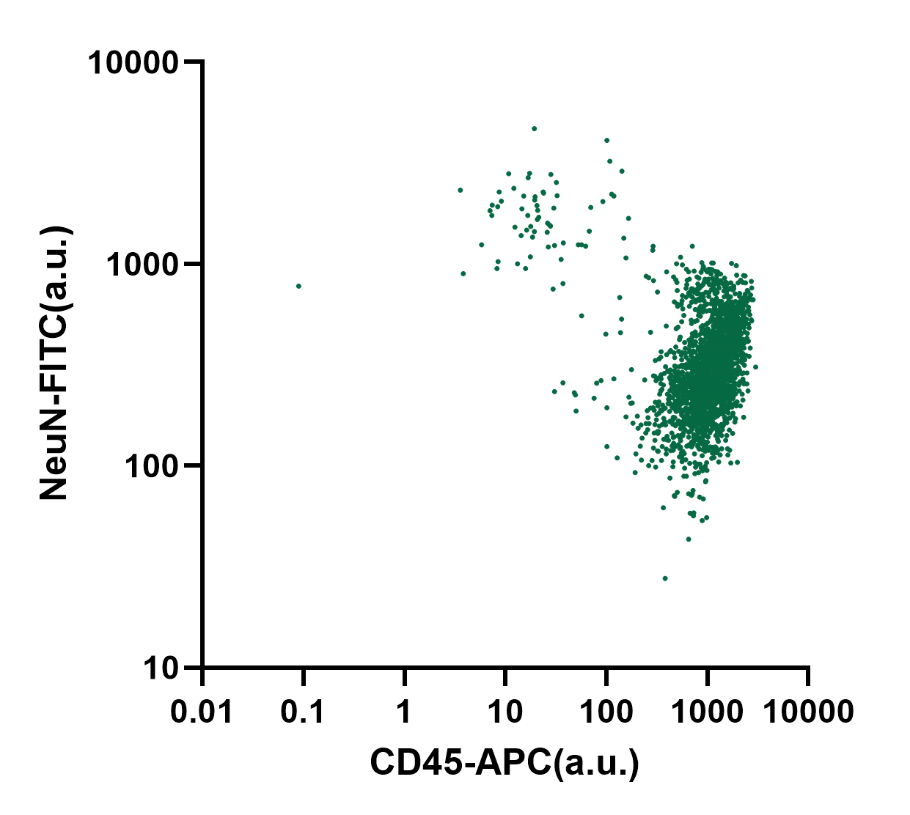


**Figure S14.** The plot 2D scatter plots of NeuN expression of CD45− and CD45+ cell populations in a peripheral blood sample taken of human patient.


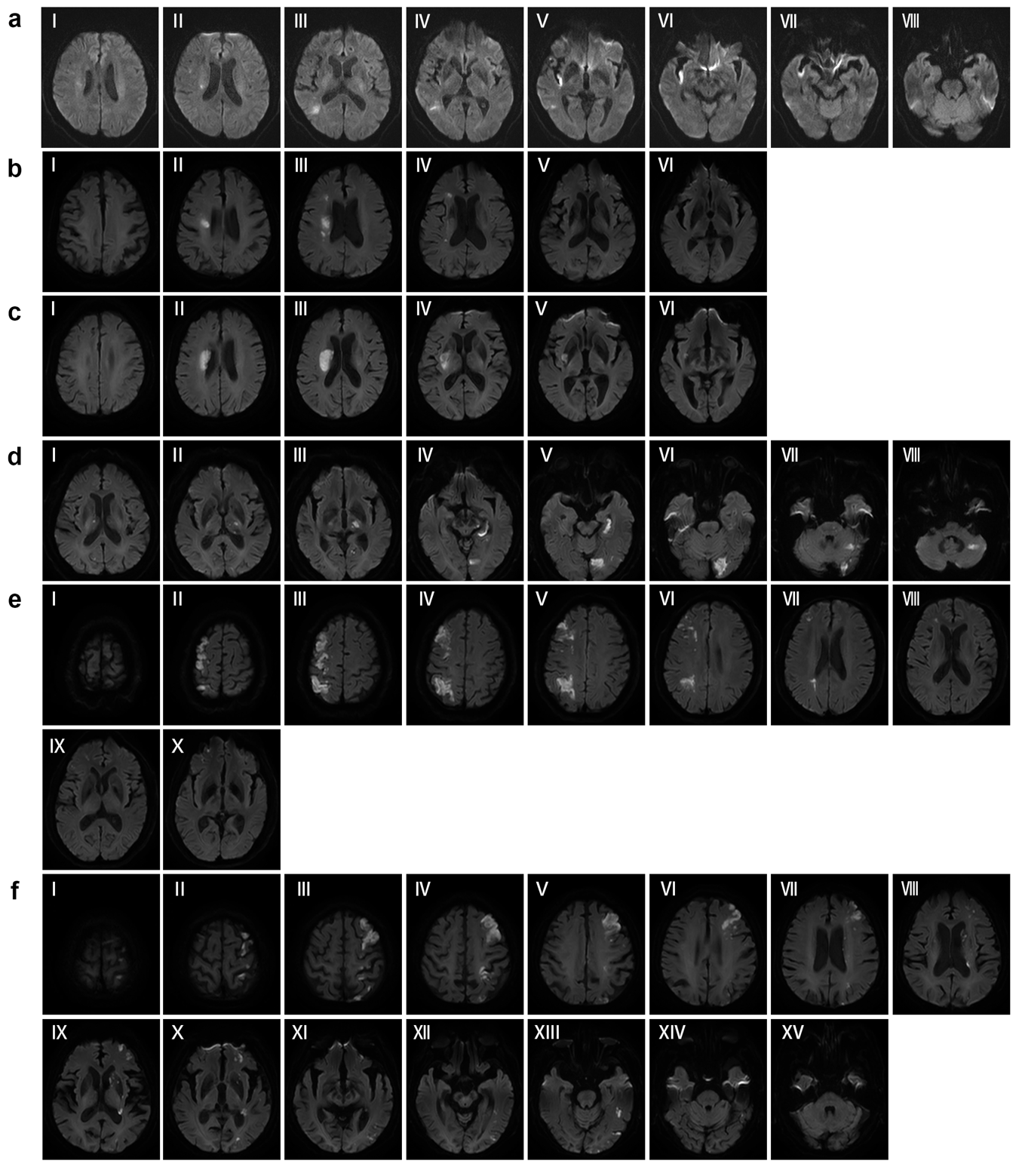


**Figure S 15. The representative MRI images from different degrees of cerebral infarction.** (**a-c**) MRI diffusion-weighted imaging of representative patients from mild group. (**d-f**) MRI diffusion-weighted imaging of representative patients from intermediate group.

**
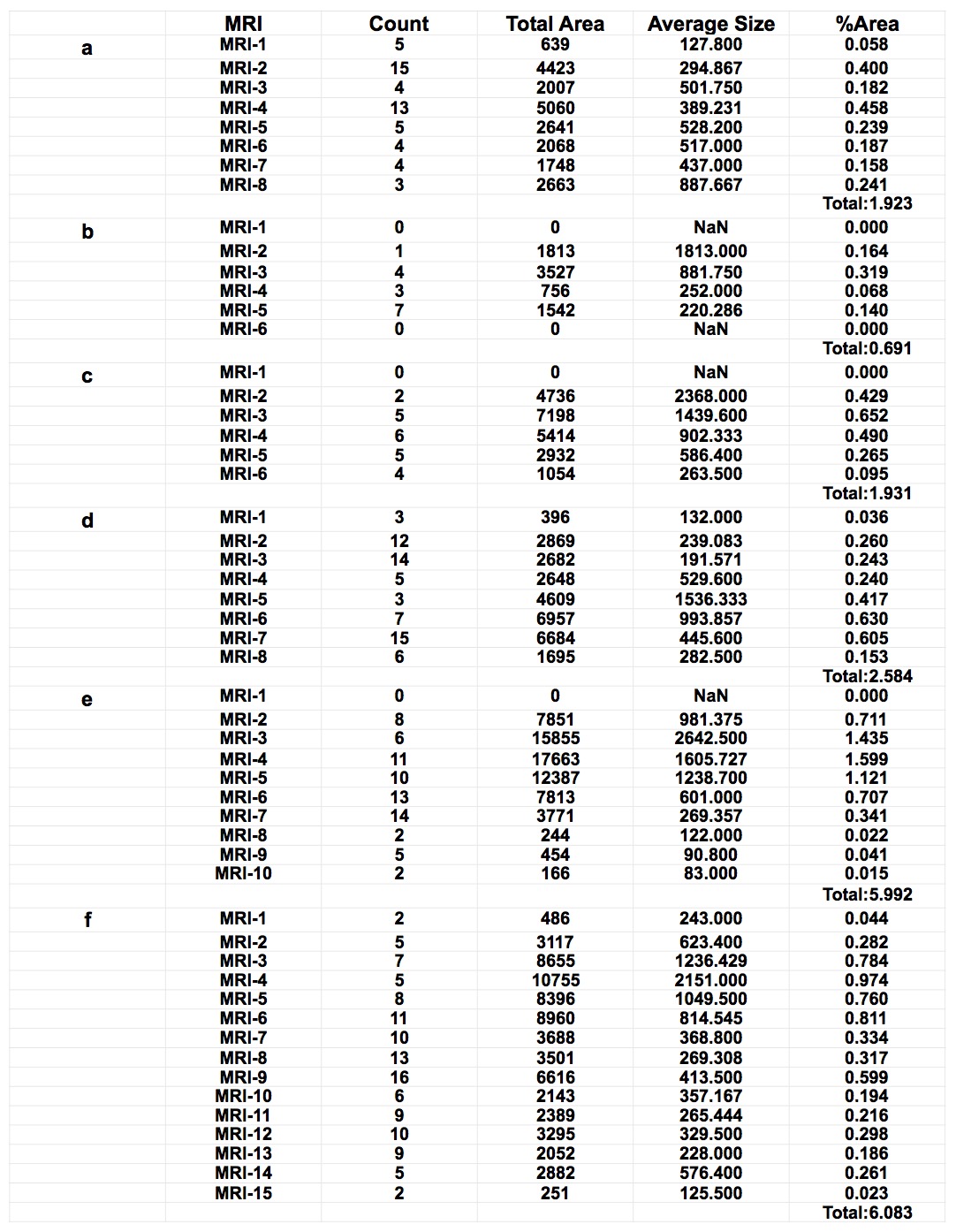
**

**Figure S16. The proportion of the cerebral infarction area in MRI images.** (**a-c**) The proportion of the cerebral infarction area in MRI images from mild group. (**d-f**) The proportion of the cerebral infarction area in MRI images from intermediate group.

**
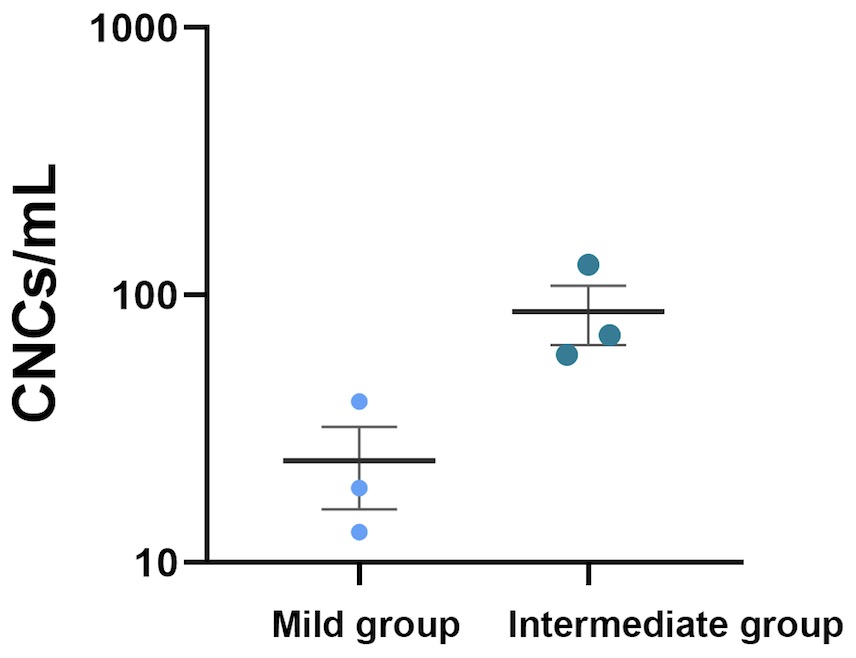
**

**Figure S17. Statistical correlation between CNCs enumeration and extent of stroke between mild group and intermediate group patients.** X-axis represents the proportion of the cerebral infarction area in MRI images. P value < 0.01.

**
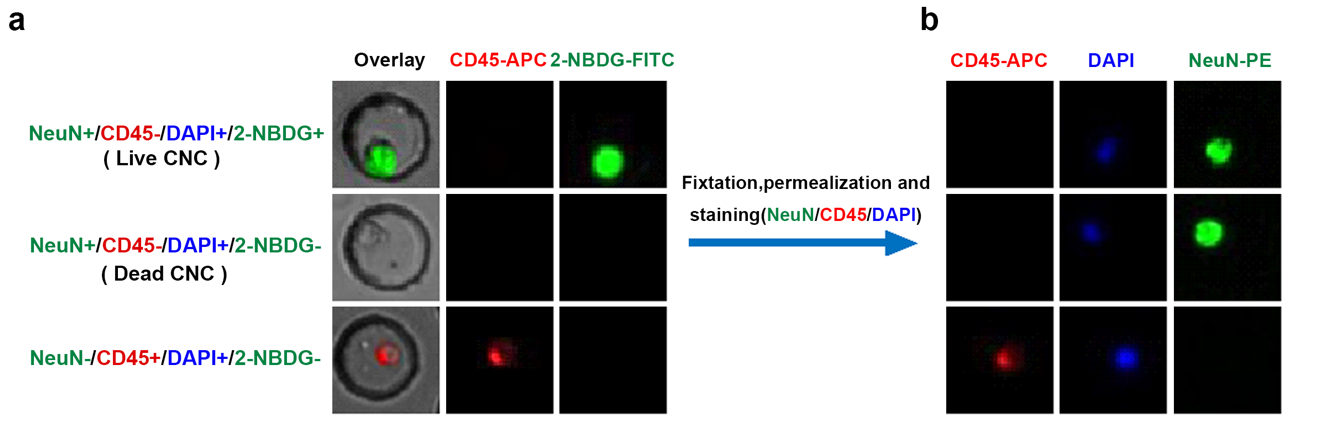
**

**Figure S18.** **Representative images of cells with NeuN^+^/CD45^-^/DAPI^+^/2-NBDG^+^ phenotype identified in peripheral blood sample of ischemic stroke patient.** **a.** Representative images of the live CNC stained with 2-NBDG-FITC (top panel), dead CNC (middle panel) and leukocytes expressing CD45-APC (bottom panel). b. Representative images of living CNC stained with NeuN-PE (top panel), dead CNC stained with NeuN-PE (middle panel) and leukocyte expressing CD45-APC (bottom panel).

**Table S1.** Pathological characteristics and CNCs enumeration of clinical samples.

| Stroke patient | Age | Gender | MRI | NeuN^+^/CD45^-^cells/mL |
| --- | --- | --- | --- | --- |
| 1 | **58** | **M^a)^** | **L-m** | **34** |
| 2 | **67** | **M** | **R-a, b** | **13** |
| 3 | **77** | **M** | **L-a** | **82** |
| 4 | **61** | **M** | **R-b, d** | **89** |
| 5 | **50** | **M** | **L-a, b, d** | **130** |
| 6 | **50** | **M** | **R-b** | **90** |
| 7 | **41** | **M** | **R-b** | **299** |
| 8 | **63** | **M** | **L-a** | **28** |
| 9 | **71** | **F** | **L-a, d** | **13** |
| 10 | **72** | **M** | **f** | **157** |
| 11 | **73** | **F** | **R-b** | **19** |
| 12 | **58** | **M** | **L-a, b** | **22** |
| 13 | **89** | **M** | **L-a** | **96** |
| 14 | **87** | **M** | **L-a, b, d** | **49** |
| 15 | **82** | **F** | **R-b, d** | **40** |
| 16 | **61** | **F** | **l^b)^** | **178** |
| 17 | **82** | **F** | **L-a** | **93** |
| 18 | **75** | **M** | **R-a** | **71** |
| 19 | **70** | **F** | **B-b, c, d** | **60** |
| 20 | **85** | **M** | **R-a; L-b** | **122** |
| 21 | **77** | **M** | **R-a** | **79** |
| 22 | **70** | **M** | **R-a** | **31** |
| 23 | **62** | **M** | **R-d; L-a** | **32** |
| 24 | **52** | **M** | **L-e** | **41** |
| 25 | **67** | **M** | **L-a, d** | **41** |
| Negative control |  |  |  |  |
| 1 | **54** | **M** | **/^b)^** | **1** |
| 2 | **48** | **F** | **/** | **0** |
| 3 | **58** | **M** | **/** | **3** |
| 4 | **58** | **M** | **/** | **1** |
| 5 | **53** | **F** | **/** | **4** |
| 6 | **45** | **F** | **/** | **10** |
| 7 | **49** | **F** | **/** | **2** |
| 8 | **65** | **M** | **/** | **1** |
| 9 | **71** | **F** | **/** | **2** |
| 10 | **49** | **F** | **/** | **2** |
| 11 | **62** | **M** | **/** | **3** |
| 12 | **63** | **M** | **/** | **6** |
| 13 | **50** | **M** | **/** | **4** |
| 14 | **73** | **F** | **/** | **6** |
| 15 | **54** | **M** | **/** | **6** |
| 16 | **46** | **F** | **/** | **6** |
| 17 | **37** | **M** | **/** | **6** |
| 18 | **79** | **M** | **/** | **2** |
| 19 | **40** | **F** | **/** | **2** |
| 20 | **64** | **F** | **/** | **0** |
| 21 | **30** | **F** | **/** | **4** |
| 22 | **28** | **M** | **/** | **7** |
| 23 | **40** | **F** | **/** | **5** |
| 24 | **55** | **M** | **/** | **5** |
| 25 | **55** | **M** | **/** | **4** |
| 26 | **27** | **F** | **/** | **1** |
| 27 | **30** | **M** | **/** | **1** |
| 28 | **55** | **M** | **/** | **7** |
| 29 | **81** | **F** | **/** | **3** |
| 30 | **54** | **F** | **/** | **1** |
| 31 | **29** | **F** | **/** | **0** |
| 32 | **72** | **M** | **/** | **4** |
| 33 | **64** | **F** | **/** | **2** |
| 34 | **29** | **F** | **/** | **0** |
| 35 | **50** | **F** | **/** | **2** |
| 36 | **50** | **F** | **/** | **3** |
| 37 | **78** | **M** | **/** | **8** |
| 38 | **63** | **M** | **/** | **7** |

^a)^ Abbreviations: NIHSS, National Institute of Health Stroke Scale; M, male; F, female; R, right; L, left; B, bilateral; ^b)^ a, temporal occipital lobe; b, centrum semiovale; c, hippocampus; d, basal ganglia; e, pons; f, medulla oblongata; g, occipital lobe; h, thalamus; i, cerebellum; j, cortex; k, hypothalamus; l, no new lesion; m, middle cerebral artery blood supply area; n, brain atrophy; o, atherosclerotic cerebral infarction; /, symptomless.

**Table S2.** Clinical and pathological data of the stroke patients

| Stroke patients | Follow-up | Hypertension | Diabetes | Atrial fibrillation | Hyperlipemia | Hyperhomocysteinemia | Smoking | Alcohol | Thrombolytic | Putian drinking water test | GCS |
| --- | --- | --- | --- | --- | --- | --- | --- | --- | --- | --- | --- |
| 1 | **D^a)^** | **+++** | **Ⅱ^b)^** | **/** | **/** | **+** | **+** | **H** | **/** | **2** | **15** |
| 2 | **W** | **/^b)^** | **/** | **++** | **/** | **/** | **H^b)^** | **/** | **/** | **2** | **15** |
| 3 | **BL** | **+++** | **/** | **/** | **/** | **/** | **/** | **/** | **/** | **1** | **4** |
| 4 | **W** | **+++** | **/** | **/** | **/** | **/** | **H** | **/** | **/** | **5** | **15** |
| 5 | **W** | **+++** | **Ⅱ** | **/** | **/** | **+** | **+** | **/** | **+** | **1** | **15** |
| 6 | **N/A** | **/** | **/** | **/** | **/** | **/** | **H** | **/** | **/** | **2** | **15** |
| 7 | **W** | **+^b)^** | **/** | **/** | **+** | **/** | **/** | **/** | **/** | **1** | **15** |
| 8 | **W** | **+++** | **Ⅱ** | **/** | **+** | **/** | **/** | **+** | **/** | **1** | **15** |
| 9 | **BL** | **+++** | **/** | **+** | **/** | **/** | **/** | **/** | **/** | **1** | **15** |
| 10 | **BL** | **+++** | **Ⅱ** | **/** | **/** | **/** | **+** | **+** | **/** | **1** | **15** |
| 11 | **N/A** | **+++** | **/** | **/** | **+** | **+** | **/** | **/** | **/** | **3** | **15** |
| 12 | **SS** | **+++** | **/** | **/** | **/** | **/** | **/** | **/** | **/** | **1** | **15** |
| 13 | **W** | **+++** | **Ⅱ** | **+** | **/** | **/** | **/** | **/** | **/** | **2** | **15** |
| 14 | **D** | **/** | **/** | **/** | **/** | **+** | **/** | **/** | **/** | **1** | **13** |
| 15 | **BL** | **+++** | **+** | **/** | **+** | **/** | **/** | **/** | **/** | **4** | **15** |
| 16 | **N/A** | **++** | **Ⅱ** | **/** | **/** | **/** | **/** | **/** | **/** | **N/A** | **N/A** |
| 17 | **D** | **++** | **/** | **/** | **+** | **/** | **/** | **/** | **/** | **3** | **7** |
| 18 | **W** | **+++** | **/** | **/** | **/** | **/** | **+** | **/** | **/** | **1** | **15** |
| 19 | **W** | **/** | **/** | **/** | **+** | **/** | **/** | **/** | **/** | **3** | **15** |
| 20 | **N/A** | **+++** | **/** | **/** | **/** | **/** | **/** | **/** | **/** | **5** | **10** |
| 21 | **W** | **+++** | **Ⅱ** | **/** | **+** | **/** | **/** | **+** | **/** | **1** | **15** |
| 22 | **SS** | **+++** | **/** | **/** | **/** | **/** | **+** | **+** | **+** | **2** | **15** |
| 23 | **W** | **+++** | **/** | **/** | **/** | **+** | **+** | **+** | **/** | **2** | **15** |
| 24 | **BL** | **++** | **Ⅱ** | **/** | **+** | **/** | **/** | **/** | **/** | **1** | **15** |
| 25 | **N/A** | **+++** | **/** | **/** | **/** | **+** | **/** | **/** | **/** | **1** | **15** |

^a)^ Abbreviations: D, dead; W, well; BL, bad quality of life; SS, secondary stroke; N/A, no available; ^b)^ Ⅱ, type 2 diabetes; +, symptoms, the number of which represents severity; /, symptomless; H, have a previous history.
